# Compulsory Immunization Protects Against Infection: What Law and Society Can Do

**DOI:** 10.20411/pai.v5i1.344

**Published:** 2020-01-20

**Authors:** Maxwell J. Mehlman, Michael M. Lederman

**Affiliations:** 1 Case Western Reserve University School of Law; Cleveland, Ohio; 2 Case Western Reserve University School of Medicine; Cleveland, Ohio

**Keywords:** immunization, immunization law, herd immunity, compulsory vaccination, childhood vaccination, anti-vaccination, anti-vaxers, measles

## Abstract

Since their broad implementation, immunizations have decreased morbidity and mortality due to a number of serious infectious diseases. In recent years, exaggerated concerns about the safety of immunizations have resulted in decreased immunization coverage in many regions and epidemic outbreaks of serious transmissible diseases – most particularly measles. This commentary reviews the legal justification for compulsory immunization and the ethical justification for civil incentives to assure compliance with immunization practices.

The concept that host defenses could be mobilized to protect against serious infectious disease was recognized in China and practiced by variolation as early as the 15^th^ century, wherein intramucosal and later intradermal inoculation of fluid from smallpox (variola) pustules would produce a relatively milder infection that would protect against the more severe naturally acquired infection. Nonetheless, infection induced by variolation could be severe, and this practice was replaced by the work of Edward Jenner in the 1800s who, at the end of the 18th century, showed that intradermal inoculation using a related pox virus–cowpox (vaccinia) that typically produced only local inflammation after intradermal inoculation also protected against smallpox infection but with a much lower risk of morbid consequences than was seen after variolation with smallpox virus.

Since then, principles of immunization (somewhat loosely called “vaccination”) have been applied to the development of numerous “vaccines” to prevent a variety of transmissible viral and bacterial infections. As a result of these practices, smallpox has been eradicated and other life-threatening transmissible infections like polio, measles, mumps, rubella, pertussis, and rabies are diminishing in many parts of the world, and morbid complications of infections like tetanus are preventable.

Many of these preventable infections are transmitted from person to person while some such as rabies are typically transmitted to humans from an animal host; however, tetanus is not transmitted from person to person. The success of immunization strategies to protect a community from person to person transmission of infection depends both on each immunized individual’s resistance to infection and also on the concept of “herd immunity” wherein even small numbers of unimmunized individuals benefit from the fact that immunized persons surrounding them are protected from infection, and this also allows them to escape exposure to the infectious agent. But herd immunity depends on a large immunized and protected “herd”, and when the proportions of immunized and protected persons fall to low enough levels, the protection to others conferred by community resistance diminishes.

Herd immunity also protects persons who cannot be protected by immunizations. Such persons include those with underlying disorders of immunity that place them at higher risk for complications of immunization, those who have hypersensitivity or allergy to components of the vaccine, and very young infants whose immune status does not yet allow them to acquire protective immunity after immunizations. Importantly, herd immunity also protects individuals who chose not to be immunized themselves, but who benefit from the compliance of their neighbors.

Immunization requirements are an exercise of broad police powers that the US Constitution grants largely to states to protect the public health. The leading case is *Jacobson v Massachusetts*, in which the US Supreme Court in 1905 upheld a Cambridge ordinance that required smallpox vaccinations and fined holdouts [1]. In the 1922 case *Zucht v King*, the Supreme Court upheld a Texas law requiring children to be vaccinated in order to attend public or private schools [2]. Today, all states require immunization for school attendance for both private and public schools. Thirty-three states have an immunization requirement for post-secondary education. Typical requirements to attend school include immunizations to prevent transmissible infections that result in diphtheria, pertussis, polio, measles, mumps, rubella, varicella, hepatitis B, hepatitis A, and Haemophilus influenza type b, as well as tetanus [3]. Parents’ failure to immunize their children also can constitute abuse or neglect [4]. The menu of required immunizations varies by state, and all required vaccines (excepting tetanus) protect against infections that are transmitted from person to person. As newer vaccines are developed, public health experts must determine if or when they should be required, balancing risks, efficacy, and state of knowledge. As an example, a few states also require immunization to prevent human papilloma virus (HPV) infection while none require immunization to prevent influenza that typically requires annual immunization to offer protection. A recently approved vaccine that effectively prevents deadly Ebola virus disease has been provided on a compassionate basis to slow epidemic spread [5]. It is too early to know if any public health authorities in epidemic regions are prepared to mandate immunization of persons at risk.

Many other countries require immunizations in order to attend school, and many also provide them free of charge. Australia requires immunization for a child to attend day care and for parents to be eligible to receive certain government benefits [6]. Italy fines parents who fail to immunize their children, and Germany recently decided to fine parents who do not provide measles immunization to their children. In Canada, Ontario requires individuals asserting a religious or philosophical exemption to complete an education session at their local public health unit that covers basic information about immunization, its safety, importance for community health, and the law, but only Ontario and New Brunswick require immunizations for school attendance [7].

The only limit placed by the US Supreme Court on state actions in *Jacobson* is that they cannot be “arbitrary or unreasonable” or “cruel or inhuman.” As an example, the government cannot require immunization if it would “seriously impair … health or probably cause … death.” This limitation arguably is the legal justification for the medical exemption allowed in all states, based on a list of recognized contraindications to immunization compiled by the CDC [8].

In addition to medical exemptions, however, 46 states allow religious exemptions. (The 4 states that do not are California, Mississippi, West Virginia, and since June 13, 2019, New York; Maine will drop its religious exemption in 2021.) There is no requirement in either the US Constitution or state constitutions that states must provide exemptions from immunization for religious reasons [9, 10]. The only judicial decisions addressing religious exemptions have held that a state cannot restrict religious exemptions to persons who belong to “recognized” religions because the government should not be determining which religions are or are not “recognized” [11-13]. At the same time, a court has allowed a state to limit religious exemptions to those who hold “genuine and sincere” religious beliefs [14].

Fifteen states also grant exemptions from immunization for philosophical or personal beliefs [15]. Yet courts have ruled that states are not required to grant such exemptions, and have denied parents’ claims that states that do not grant them violate constitutional rights [2, 16, 17].

## Can the Law Do More?

Clearly, then, states can limit exemptions to those for sound medical reasons. Can the law do more? Following the lead of Australia, state legislatures and Congress could make immunization a condition for adults and children to obtain health benefits under Medicare, Medicaid, and even from private insurers, with exemptions only for sound medical reasons. In addition, Congress could condition federal funding for states on states tightening immunization requirements [18].

In the past, states quarantined, isolated, and fined citizens suspected of being infected or who had not been immunized [19]. New York began imposing fines in April 2019 in response to a measles outbreak, and a state court upheld the fines against parental objections [20]. The problem with fines, however, is that they especially burden individuals and families with low incomes and, in the case of parental refusals to immunize their children, thereby punish the children as well as their parents.

## Can States Mandate Immunization?

The decision in *Jacobson* supports mandatory immunization of children and adults to prevent or mitigate serious outbreaks of transmissible diseases. The only limit on state action, as the Court in *Jacobson* recognized, is that it cannot be arbitrary or unreasonable; for example, the state must have an adequate medical justification for its actions.

Some commentators contend that the state cannot immunize people without their permission, citing cases such as *Quinlan* and *Cruzan* that hold that competent patients cannot be treated for medical conditions against their will [21]. Reliance on these cases is misplaced however; they do not involve transmissible diseases, and the harm, if any, is to the patient herself. In contrast, the failure to be immunized impairs herd immunity, which protects not only persons who choose not to be vaccinated, but those who cannot be immunized for medical reasons, those who are too young to be immunized, and those who received immunizations but either did not mount an adequate immune response or whose immunity has waned over time [22].

Others say that there is no need for mandatory immunization, since public health authorities can quarantine those who refuse to be immunized until they relent [23]. But the alternative of quick, inexpensive, compulsory immunization is much less costly than incarceration at public expense, and in the case of children, the public is unlikely to support confining children for prolonged periods when parents refuse to consent to immunization.

## Can Society Do More?

If it is in society’s interest to assure broad population immunity to prevent transmissible diseases, civil society could do more. Educational efforts are often met with profound resistance by persons opposed to immunization who have mobilized social media to support their perspectives and propaganda [24]. Civil society must invest in efforts to counter disinformation, perhaps by redirecting searches to effective advertisements much like an organization called Moonshot redirects internet searches for white supremacist sites [25]. Additional incentives could be introduced. Individuals who become ill after exposure to persons who refused immunization could bring civil actions against them, or their parents, seeking compensation for their losses [26]. Proving that a specific unimmunized individual caused the plaintiff’s illness is likely to be difficult and would require not only clear evidence of exposure but also careful examination of the responsible microbe isolated from both persons and with sufficient genetic homologies between the pathogens to assure relatedness. Moreover, low-income families refusing to be immunized would be unlikely to be able to fully compensate their victims.

Physicians and other ambulatory health care practices could, if sustained efforts at persuasion fail, refuse to allow unimmunized patients into their practice to protect their other patients who could risk exposure to infection in a crowded waiting room. Even without the government legislation discussed above, private insurance carriers could deny benefits to individuals and families who refuse required immunizations; denial could be specifically for benefits to treat infections covered by the immunizations or could be broader. During outbreaks of airborne transmissible disease, crowded facilities such as airplanes could deny access to the unimmunized. Other venues that accommodate large numbers of attendees such as amusement parks and sports arenas also could require documentation of immunization, and in these settings, the magnitude of the epidemic and attendant risk would have to justify the burden of documentation.

In our view, both legal precedent and medical science support compulsory immunization of children and adults except where medically contraindicated. Adults have the right to refuse medical interventions, including immunization, but this right can be overridden to achieve the compelling public health objective of producing herd immunity against sufficiently serious infectious diseases. This was the case recently in Samoa where a measles epidemic that caused more than 5,000 cases and more than 80 deaths was met by a declaration of emergency and mandatory immunization [27]. In the case of children, the benefit to the child from being immunized alone justifies this position, even without considering the benefit to the public from enhancing herd immunity.

## Can Refusal to immunize Be Justified When the Population has Achieved Herd Immunity?

It might be objected that herd immunity can be reached without immunizing an entire population, and since immunizations can, with varying but very low frequencies, cause serious morbidity, one could argue that adults should be able to refuse immunization for themselves and their children when sufficient herd immunity is achieved. Thus, once the public health interest is satisfied by herd immunity, do individuals have the right to compare and hold in balance their very low risks from immunization with the very low risks of infection if not immunized?

It must be recognized that herd immunity is not absolute, and the proportions of immunized and protected persons in a population necessary to provide herd immunity will vary according to the transmissibility of the pathogen, susceptibility of the population to the pathogen, and the effectiveness of the immunization [28]. Although herd immunity can in time result in eradication of a transmissible infection (as has been the case for smallpox), until the disease is eradicated, a greater proportion of immunized persons will, particularly as natural infection diminishes, decrease the proportion of persons at risk in the population, thereby enhancing the protection of others [29]. Thus, until infection is eradicated, the sufficiency of herd immunity is difficult to define, and greater breadth of immunization increasingly serves the interest of public health. Moreover, on this variable and moving scale of infection risk there is no generally accepted method for balancing the potential benefits and risks of immunizations to any particular individual. It is also not clear what system of ethics refusers can apply to a stance wherein they benefit from the substantial protection afforded by the immunization of others while refusing to partake in the very low risks that immunization entails.

## CONCLUSION

As the risk of infection diminishes with greater public compliance with immunization policies, compulsory immunization programs are rendered both necessary for the public health and vulnerable to complaint by individuals who demand preferential protection from risk. Society should develop and apply effective strategies to counter the misinformation that is widespread on the internet and given credibility by those reluctant to be immunized. Society through legal precedent and civil incentives should promote compulsory immunization programs with only limited exemptions for persons with medical contraindications to a vaccine.
